# Supplementary material for: Campylobacter jejuni transcriptome changes during loss of culturability in water
Source: PLoS One. 2017 Nov 30;12(11):e0188936. doi: 10.1371/journal.pone.0188936 (PMC5708674; doi:10.1371/journal.pone.0188936)
Supplement: S2 Table — Cpm displayed are normalised by the Trimmed Mean of M-values (TMM) method, implemented in the edgR Bioconductor package. In brackets the log-fold-change cpm relative to the MHB is shown. Cells are shaded according to log-fold-change (yellow = negative, green = positive). (DOCX) [file pone.0188936.s002.docx]

**S2 Table.** Table summarizing genes that are up or downregulated ≥ 2-fold compared to the MHB control in read counts per million (cpm) across all three experimental conditions. Cpm displayed are normalised by the Trimmed Mean of M-values (TMM) method, implemented in the edgR Bioconductor package. In brackets the log-fold-change cpm relative to the MHB is shown. Cells are shaded according to log-fold-change (yellow=negative, green=positive).

| **Gene_ID** | **ID** | **MHB Ctrl CPM average** | **Time 0 CPM average [FC to MHB; p-value]** | **25°C/24h CPM average [FC to MHB; p-value]** | **4°C/72h CPM average [FC to MHB; p-value]** |
| --- | --- | --- | --- | --- | --- |
| ≥ 2-fold upregulated compared to the control | | | | | |
| CJM1_1553 | *chuC* | 0.16 | 35.37 [7.7; p=0.00608] | 3.39 [5.02; p=0.23979] | 2 [3.85; p=0.00608] |
| CJM1_0057 | CJM1_0057 | 0.28 | 48.48 [7.37; p=0.01233] | 4.78 [4.74; p=0.23192] | 2.78 [3.56; p=0.01233] |
| CJM1_0713 | CJM1_0713 | 0.69 | 68.58 [6.62; p=0.01039] | 10.57 [4.66; p=0.13293] | 6.69 [3.56; p=0.01039] |
| CJM1_1551 | *chuA* | 2.93 | 287.47 [6.6; p=0.01483] | 25.09 [3.81; p=0.20271] | 26.17 [3.45; p=0.01483] |
| CJM1_0905 | CJM1_0905 | 1.28 | 119.29 [6.52; p=0.02358] | 10.14 [3.7; p=0.18353] | 11.95 [3.51; p=0.02358] |
| CJM1_0720 | CJM1_0720 | 1.87 | 163.43 [6.43; p=0.03891] | 10.93 [3.25; p=0.27079] | 12.49 [3.03; p=0.03891] |
| CJM1_0719 | CJM1_0719 | 0.68 | 50.61 [6.18; p=0.00461] | 5.76 [3.78; p=0.19032] | 7.29 [3.7; p=0.00461] |
| CJM1_0056 | CJM1_0056 | 0.35 | 26.1 [6.17; p=0.0262] | 17.05 [6.32; p=0.05159] | 7.58 [4.69; p=0.0262] |
| CJM1_0146 | CJM1_0146 | 2.06 | 142.99 [6.1; p=0.00671] | 13.59 [3.44; p=0.17457] | 20.8 [3.63; p=0.00671] |
| CJM1_0714 | CJM1_0714 | 1.62 | 109.26 [6.06; p=0.02156] | 23.6 [4.59; p=0.11425] | 13.84 [3.38; p=0.02156] |
| CJM1_1668 | CJM1_1668 | 0.17 | 11.31 [6.02; p=0.01393] | 1 [3.25; p=0.27212] | 0.98 [2.8; p=0.01393] |
| CJM1_1636 | CJM1_1636 | 0.51 | 29.78 [5.84; p=0.00977] | 2.19 [2.8; p=0.23718] | 4.11 [3.29; p=0.00977] |
| CJM1_0906 | CJM1_0906 | 1.69 | 95.24 [5.79; p=0.01625] | 9.23 [3.17; p=0.15919] | 10.21 [2.88; p=0.01625] |
| CJM1_1286 | CJM1_1286 | 0.75 | 39.33 [5.68; p=0.01921] | 3.12 [2.77; p=0.14402] | 6.04 [3.29; p=0.01921] |
| CJM1_1637 | CJM1_1637 | 2.52 | 81.97 [5; p=0.04207] | 10.78 [2.85; p=0.05491] | 11.33 [2.46; p=0.04207] |
| CJM1_1665 | CJM1_1665 | 1.89 | 30.48 [3.99; p=0.01917] | 5.29 [2.24; p=0.01662] | 6.97 [2.18; p=0.01917] |
| CJM1_1346 | *katA* | 99.9 | 1138.48 [3.5; p=0.00028] | 758.67 [3.7; p=0.00005] | 678.25 [3.06; p=0.00028] |
| CJM1_1559 | CJM1_1559 | 0.11 | 1.27 [3.49; p=0.02954] | 0.41 [2.62; p=0.14676] | 0.45 [2.32; p=0.02954] |
| CJM1_0670 | *flgH* | 1114.4 | 11689.69 [3.38; p=0.0064] | 9762.05 [3.91; p=0.00076] | 17666.61 [4.28; p=0.0064] |
| CJM1_0972 | CJM1_0972 | 336.29 | 3542.16 [3.38; p=0.00696] | 2251.04 [3.52; p=0.00079] | 3432.35 [3.65; p=0.00696] |
| CJM1_1181 | CJM1_1181 | 77.81 | 673.15 [3.1; p=0.0012] | 1496.14 [5.04; p=0.00125] | 926.26 [3.87; p=0.0012] |
| CJM1_0679 | *flgF* | 757.77 | 6261.01 [3.03; p=0.0038] | 6719.3 [3.92; p=0.00057] | 8915.93 [3.85; p=0.0038] |
| CJM1_0503 | *flgB* | 549.27 | 4440.36 [3; p=0.0088] | 5079.02 [3.98; p=0.00169] | 7893.27 [4.14; p=0.0088] |
| CJM1_0023 | CJM1_0023 | 71.6 | 564.2 [2.96; p=0.00041] | 1206.66 [4.85; p=0] | 935.64 [4; p=0.00041] |
| CJM1_1480 | CJM1_1480 | 12.92 | 100.93 [2.95; p=0.00261] | 44.72 [2.56; p=0.0047] | 80.79 [2.94; p=0.00261] |
| CJM1_0051 | *flgD* | 1055.54 | 8038.4 [2.92; p=0.00226] | 8185.74 [3.73; p=0.00162] | 12358.99 [3.85; p=0.00226] |
| CJM1_1672 | *leuC* | 61.9 | 472.19 [2.92; p=0.00545] | 273.71 [2.91; p=0.01326] | 217.15 [2.11; p=0.00545] |
| CJM1_1408 | *flgI* | 515.67 | 3663.56 [2.82; p=0.00552] | 3958.36 [3.72; p=0.00153] | 5356.66 [3.67; p=0.00552] |
| CJM1_1183 | *metE* | 92.36 | 651.76 [2.81; p=0.00112] | 1651.04 [4.93; p=0.00021] | 1042.61 [3.79; p=0.00112] |
| CJM1_1182 | CJM1_1182 | 37.7 | 261.9 [2.78; p=0.0015] | 407.39 [4.21; p=0.00068] | 420.17 [3.77; p=0.0015] |
| CJM1_0064 | CJM1_0064 | 116.45 | 783.12 [2.74; p=0.01418] | 320.11 [2.23; p=0.00429] | 886.73 [3.22; p=0.01418] |
| CJM1_0698 | CJM1_0698 | 518.7 | 3389.37 [2.69; p=0.0055] | 3526.54 [3.54; p=0.00014] | 3181.67 [2.91; p=0.0055] |
| CJM1_1673 | *leuB* | 38.13 | 246.72 [2.68; p=0.00733] | 169.43 [2.92; p=0.00638] | 124.25 [2; p=0.00733] |
| CJM1_1275 | *flmA* | 755.85 | 4626.2 [2.6; p=0.00214] | 6525.44 [3.89; p=0.00073] | 6449.72 [3.39; p=0.00214] |
| CJM1_1224 | CJM1_1224 | 1478.54 | 8739.3 [2.55; p=0.01626] | 10881.79 [3.66; p=0.0033] | 9934.76 [3.04; p=0.01626] |
| CJM1_0961 | CJM1_0961 | 49.41 | 283.15 [2.5; p=0.00078] | 456.74 [3.98; p=0.0004] | 319.32 [2.99; p=0.00078] |
| CJM1_0282 | *modA* | 47.64 | 267.32 [2.47; p=0.00647] | 388.41 [3.8; p=0.00008] | 394.59 [3.35; p=0.00647] |
| CJM1_0952 | CJM1_0952 | 403.34 | 2263.36 [2.47; p=0.01241] | 1838.55 [2.96; p=0.00215] | 2826.02 [3.11; p=0.01241] |
| CJM1_1573 | CJM1_1573 | 6.25 | 34 [2.43; p=0.02255] | 14.85 [2.02; p=0.00482] | 56.72 [3.48; p=0.02255] |
| CJM1_0680 | *flgG* | 1169.74 | 5886.65 [2.32; p=0.00256] | 3019.38 [2.15; p=0.0007] | 4948.63 [2.38; p=0.00256] |
| CJM1_1184 | *metF* | 18.21 | 92.06 [2.32; p=0.01069] | 60.5 [2.5; p=0.00074] | 71.19 [2.26; p=0.01069] |
| CJM1_1478 | *acsA* | 369.27 | 1732.58 [2.22; p=0.00199] | 3993.28 [4.21; p=0.00296] | 2990.27 [3.31; p=0.00199] |
| CJM1_1572 | CJM1_1572 | 96.46 | 448.99 [2.2; p=0.0067] | 335.7 [2.57; p=0.00035] | 669.43 [3.09; p=0.0067] |
| CJM1_0502 | *flgC* | 760.16 | 3392.2 [2.14; p=0.00688] | 3013.17 [2.76; p=0.00344] | 4996.89 [3.01; p=0.00688] |
| CJM1_0358 | CJM1_0358 | 206.71 | 920.17 [2.14; p=0.00262] | 763.26 [2.66; p=0.00006] | 1000.95 [2.57; p=0.00262] |
| CJM1_0005 | CJM1_0005 | 762.67 | 3369.12 [2.13; p=0.00829] | 3588.98 [3.01; p=0.00169] | 3676.32 [2.56; p=0.00829] |
| CJM1_0522 | *flaG* | 1056.06 | 4557.57 [2.1; p=0.00137] | 6300.85 [3.35; p=0.00191] | 6485.75 [2.91; p=0.00137] |
| CJM1_0197 | CJM1_0197 | 48.21 | 205.55 [2.08; p=0.00575] | 230.07 [3.03; p=0.006] | 192.47 [2.29; p=0.00575] |
| CJM1_1000 | CJM1_1000 | 1640.42 | 6792.24 [2.04; p=0.01417] | 7589.75 [2.99; p=0.00211] | 8542.99 [2.68; p=0.01417] |
| ≥ 2-fold downregulated compared to the control | | | | |  |
| CJM1_1420 | *ctsR* | 73.05 | 13.92 [-2.41; p=0.00525] | 2.53 [-4.08; p=0.00397] | 10.68 [-2.48; p=0.00525] |
| CJM1_0264 | *tal* | 325.36 | 60.4 [-2.44; p=0.00209] | 40.33 [-2.24; p=0.00075] | 47.64 [-2.48; p=0.00209] |
| CJM1_0758 | *napH* | 498.71 | 90.15 [-2.48; p=0.00019] | 34.59 [-3.09; p=0.00007] | 49.25 [-3.04; p=0.00019] |
| CJM1_0759 | *napB* | 888.98 | 79.57 [-3.5; p=0.00054] | 74.79 [-2.8; p=0.0003] | 77.38 [-3.23; p=0.00054] |
| CJM1_0757 | *napG* | 1378.46 | 117.75 [-3.56; p=0.00013] | 142.99 [-2.5; p=0.00008] | 185.26 [-2.6; p=0.00013] |
