# Supplementary material for: Campylobacter jejuni transcriptome changes during loss of culturability in water
Source: PLoS One. 2017 Nov 30;12(11):e0188936. doi: 10.1371/journal.pone.0188936 (PMC5708674; doi:10.1371/journal.pone.0188936)
Supplement: S4 Table — Fold change compared to the MHB Control, as determined by edgeR, the Paired student’s T-test and PPLR determined through BitSeq analysis are shown in brackets. Stressor abbreviations: S, starvation; O, osmotic; OX, oxidative; A, aerobic; HS, heat-shock; F/T; freeze-thaw; I, iron stress; CD, cell density. (DOCX) [file pone.0188936.s004.docx]

**S4 Table.** Table showing gene expression of previously described stress response genes shown as average read counts per million (cpm). Fold change compared to the MHB Control, as determined by edgeR, the Paired student’s T-test and PPLR determined through BitSeq analysis are shown in brackets. Stressor abbreviations: S, starvation; O, osmotic; OX, oxidative; A, aerobic; HS, heat-shock; F/T; freeze-thaw; I, iron stress; CD, cell density

| **Stressor** | **ID** | **Function** | **MHB Ctrl average CPM** | **Time 0 average CPM [FC; p-value; PPLR]** | **25degrees/24hrs average CPM [FC; p-value; PPLR]** | **4 degrees/72hrs average CPM [FC; p-value; PPLR]** |
| --- | --- | --- | --- | --- | --- | --- |
| S/O | *ppk* | polyphosphate kinase | 780.84 | 480.19 [-0.72; p=0.025; PPLR=0.181] | 360.93 [-0.34; p=0.002; PPLR=0.008] | 406.6 [-0.65; p=0.003; PPLR=0.018] |
| S | *pckA* | phosphoenolpyruvate carboxykinase | 2058.55 | 1018.65 [-1.03; p=0.006; PPLR=0.982] | 1125.38 [-0.1; p=0.018; PPLR=0.056] | 1134.59 [-0.56; p=0.005; PPLR=0.755] |
| OX/A/HS | *htrA* | protease domain | 1829.43 | 1095.95 [-0.75; p=0.019; PPLR=0.03] | 1639.09 [0.62; p=0.335; PPLR=0.344] | 1409.53 [-0.08; p=0.065; PPLR=0.056] |
| S/OX/A | *spoT* | penta-phosphate guanosine-3'-pyrophosphohydrolase | 569.07 | 443.68 [-0.37; p=0.031; PPLR=0.425] | 181.32 [-0.88; p=0.001; PPLR=0.036] | 308.99 [-0.58; p=0.003; PPLR=0.37] |
| OX/A/HS | *htrB* | putative lipid A biosynthesis acyltransferase | 31.94 | 71.79 [1.15; p=0.016; PPLR=0.991] | 10.43 [-0.86; p=0.007; PPLR=0.092] | 15.05 [-0.79; p=0.016; PPLR=0.241] |
| OX/A/F/T | *sodB* | superoxide dismutase | 1389.04 | 2489.46 [0.83; p=0.001; PPLR=0.975] | 3451.25 [2.09; p=0.007; PPLR=0.999] | 3035.5 [1.42; p=0.015; PPLR=0.992] |
| OX/A/F/T | *katA* | catalase | 99.9 | 1138.48 [3.5; p=0; PPLR=1] | 758.67 [3.7; p=0; PPLR=1] | 678.25 [3.06; p=0; PPLR=1] |
| OX/A/HS | *hspR* | transcriptional regulator, MerR family | 80.59 | 99.92 [0.3; p=0.012; PPLR=0.973] | 46.92 [-0.01; p=0.005; PPLR=0.044] | 115.48 [0.82; p=0.023; PPLR=0.964] |
| OX/A | *dcuA* | anaerobic C4-dicarboxylate transporter | 2856 | 1247.95 [-1.21; p=0.034; PPLR=0.013] | 1042.04 [-0.68; p=0.015; PPLR=0.006] | 946.85 [-1.3; p=0.015; PPLR=0.002] |
| OX/A | *dps* | DNA protection during starvation protein | 2942.24 | 3666.08 [0.3; p=0.208; PPLR=0.786] | 2552.09 [0.57; p=0.02; PPLR=0.231] | 3334.12 [0.48; p=0.112; PPLR=0.716] |
| OX/A | *perR* | transcriptional regulator Fur family | 76.54 | 77.48 [0; p=0.935; PPLR=0.938] | 30.52 [-0.55; p=0.002; PPLR=0.027] | 52.2 [-0.26; p=0.023; PPLR=0.468] |
| OX/A | *sdhA* | succinate dehydrogenase flavoprotein subunit | 2424.6 | 547.53 [-2.16; p=0.001; PPLR=0.011] | 496.54 [-1.52; p=0.001; PPLR=0.003] | 554.75 [-1.83; p=0.003; PPLR=0.005] |
| OX/A | *sdhB* | succinate dehydrogenase iron-sulfur protein | 913.27 | 217.76 [-2.08; p=0.001; PPLR=0.014] | 300.37 [-0.83; p=0.001; PPLR=0.002] | 232.27 [-1.68; p=0.002; PPLR=0] |
| OX/A | *sdhC* | succinate dehydrogenase, subunit C | 742.3 | 150.47 [-2.32; p=0.002; PPLR=0.01] | 275.78 [-0.65; p=0.003; PPLR=0.006] | 195.17 [-1.63; p=0.004; PPLR=0.006] |
| OX/A | *tsaA* | probable peroxiredoxin | 1668.96 | 4793.54 [1.51; p=0.005; PPLR=0.994] | 5599.44 [2.52; p=0.001; PPLR=0.988] | 4476.18 [1.72; p=0.011; PPLR=0.996] |
| OX/A | *fdxA* | ferredoxin, 4Fe-4S | 2969.02 | 1828.71 [-0.71; p=0.026;PPLR=0.009] | 1747.21 [0.01; p=0.01;PPLR=0.029] | 2136.52 [-0.18; p=0.025; PPLR=0.042] |
| OX/A | *fdxA* | ferredoxin, 4Fe-4S | 103.63 | 78.15 [-0.42; p=0.109; PPLR=0.466] | 22.37 [-1.44; p=0.008; PPLR=0.385] | 52.39 [-0.69; p=0.028; PPLR=0.008] |
| O | CJM1_1208 | two-component sensor histidine kinase (cj1226c) | 1061.29 | 552.88 [-0.95; p=0.026;PPLR=0.138] | 87.68 [-2.83; p=0.011; PPLR=0] | 350.02 [-1.3; p=0.017; PPLR=0.011] |
| O | CJM1_0221 | mechanosensitive ion channel family protein | 399.96 | 384.88 [-0.07; p=0.406; PPLR=0.858] | 184.79 [-0.34; p=0.004; PPLR=0.104] | 238.57 [-0.45; p=0.003; PPLR=0.318] |
| O | CJM1_0980 | putative membrane protein | 322.17 | 410.84 [0.34; p=0.018; PPLR=0.965] | 144.69 [-0.38; p=0.001; PPLR=0.073] | 328.78 [0.33; p=0.011; PPLR=0.607] |
| I | *chuA* | tonB-dependent heme receptor | 2.93 | 287.47 [6.6; p=0.015; PPLR=0.999] | 25.09 [3.81; p=0.203; PPLR=0.994] | 26.17 [3.45; p=0.303; PPLR=0.996] |
| I | *chuB* | hemin ABC transporter | 19.49 | 123.28 [2.65; p=0.012; PPLR=0.994] | 11.8 [0; p=0.41; PPLR=0.512] | 33.5 [1.08; p=0.229; PPLR=0.837] |
| I | *chuC* | hemin uptake ATP-binding protein | 0.16 | 35.37 [7.7; p=0.006; PPLR=1] | 3.39 [5.02; p=0.24; PPLR=0.999] | 2 [3.85; p=0.39; PPLR=0.965] |
| I | *chuD* | hemin uptake periplasmic protein | 0.78 | 39.36 [5.64; p=0.019; PPLR=0.995] | 4.19 [3.14; p=0.182; PPLR=0.689] | 3.78 [2.56; p=0.388; PPLR=0.851] |
| I | CJM1_1550 | heme oxygenase | 65.87 | 122.85 [0.89; p=0.036; PPLR=0.951] | 96.73 [1.33; p=0.021; PPLR=0.678] | 99.22 [0.89; p=0.033; PPLR=0.755] |
| I | *fur* | ferric uptake regulation protein | 328.4 | 254.77 [-0.38; p=0.037; PPLR=0.185] | 193.68 [0.01; p=0.036; PPLR=0.007] | 287.95 [0.11; p=0.239; PPLR=0.214] |
| HS | CJM1_1347 | ankyrin-containing protein | 8.06 | 89.21 [3.46; p=0.002; PPLR=0.992] | 17.09 [1.85; p=0.007; PPLR=0.75] | 25.46 [1.96; p=0.002; PPLR=0.873] |
| HS | *clpP* | ATP-dependent Clp protease proteolytic subunit | 839.7 | 445.85 [-0.93; p=0.007; PPLR=0.008] | 381.22 [-0.37; p=0.003; PPLR=0.002] | 624.62 [-0.13; p=0.027; PPLR=0.07] |
| HS | *dnaJ* | chaperone protein | 44.77 | 147.01 [1.7; p=0.027; PPLR=0.987] | 39.05 [0.55; p=0.438; PPLR=0.886] | 48.1 [0.4; p=0.693; PPLR=0.932] |
| HS | *dnaK* | chaperone protein dnaK | 1537.72 | 1732.89 [0.16; p=0.341; PPLR=0.951] | 2096.59 [1.22; p=0.109; PPLR=0.979] | 1705.46 [0.45; p=0.049; PPLR=0.777] |
| HS | *groL* | 60 kDa chaperonin | 3801.27 | 4776.5 [0.32; p=0.03; PPLR=0.865] | 8249.38 [1.89; p=0.004; PPLR=0.996] | 5659.37 [0.87; p=0.004; PPLR=0.961] |
| HS | *groS* | 10 kDa chaperonin | 205.18 | 380.17 [0.88; p=0.009; PPLR=0.959] | 830.63 [2.79; p=0; PPLR=0.997] | 530.73 [1.67; p=0.001; PPLR=0.99] |
| HS | *grpE* | protein grpE | 49.43 | 102.53 [1.04; p=0.001; PPLR=0.995] | 118.54 [2.03; p=0.011; PPLR=0.964] | 102.37 [1.35; p=0.005; PPLR=0.928] |
| HS | *hrcA* | heat-inducible transcription repressor | 152.21 | 572.39 [1.9; p=0.007; PPLR=0.999] | 237.95 [1.42; p=0.093; PPLR=0.904] | 670.18 [2.44; p=0; PPLR=0.997] |
| HS | *hslU* | ATP-dependent hsl protease ATP-binding subunit | 1841.65 | 1291.58 [-0.52; p=0.024; PPLR=0.099] | 1247.7 [0.21; p=0.033; PPLR=0.037] | 1206.78 [-0.31; p=0.015; PPLR=0.019] |
| HS | *lon* | ATP-dependent protease | 868.77 | 737.04 [-0.25; p=0.218; PPLR=0.729] | 277.95 [-0.88; p=0.007; PPLR=0.02] | 470.73 [-0.59; p=0.014; PPLR=0.026] |
| HS | *racR* | DNA-binding response regulator | 238.19 | 162.83 [-0.56; p=0.096; PPLR=0.256] | 133.85 [-0.06; p=0.05; PPLR=0.025] | 227.81 [0.23; p=0.694; PPLR=0.427] |
| HS | *racS* | sensor protein | 134.05 | 181.55 [0.42; p=0.049; PPLR=0.856] | 30.87 [-1.37; p=0.014; PPLR=0.023] | 70.07 [-0.64; p=0.047; PPLR=0.165] |
| CD | *luxS* | autoinducer | 1424.63 | 2271.24 [0.66; p=0.02; PPLR=1] | 2556.56 [1.62; p=0; PPLR=0.999] | 2940.34 [1.34; p=0.004; PPLR=1] |
