# Supplementary figures and images for: Campylobacter jejuni transcriptome changes during loss of culturability in water
Source: PLoS One. 2017 Nov 30;12(11):e0188936. doi: 10.1371/journal.pone.0188936 (PMC5708674; doi:10.1371/journal.pone.0188936)

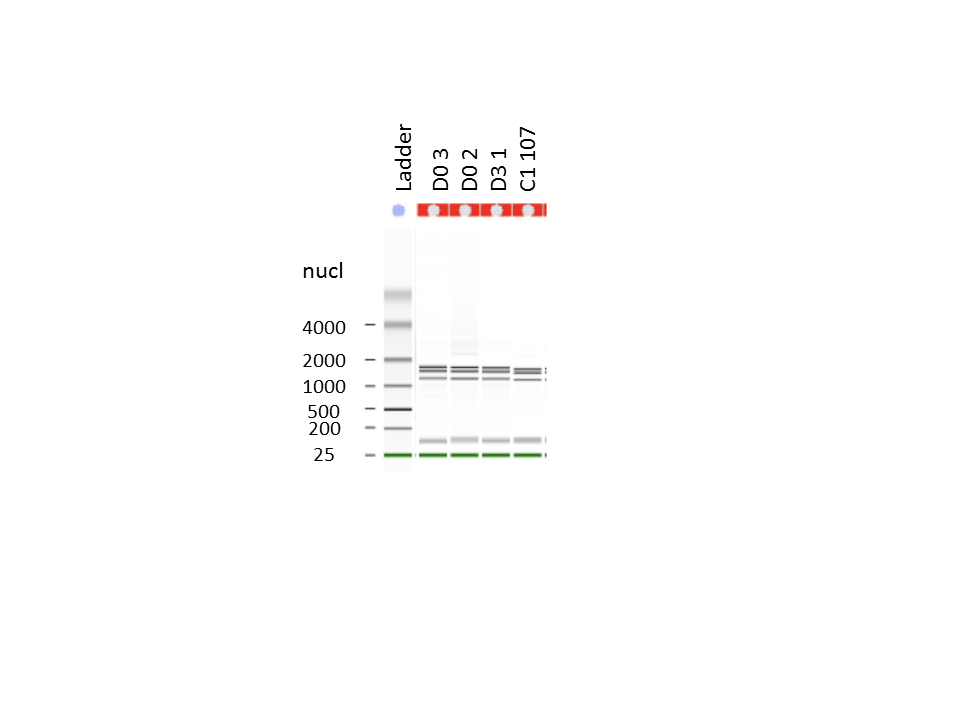

Supplement: S1 Fig — Example samples are shown for a sample from 25°C (24 h) (D1 3), Time 0 (D0 2), 4°C (72 h) (D3 1) and the Mueller Hinton Broth Control (C1 107). There was no evidence for RNA degradation in the 24 h or 72 h water samples compared to the time zero or control samples. (TIF) [file pone.0188936.s007.tif]

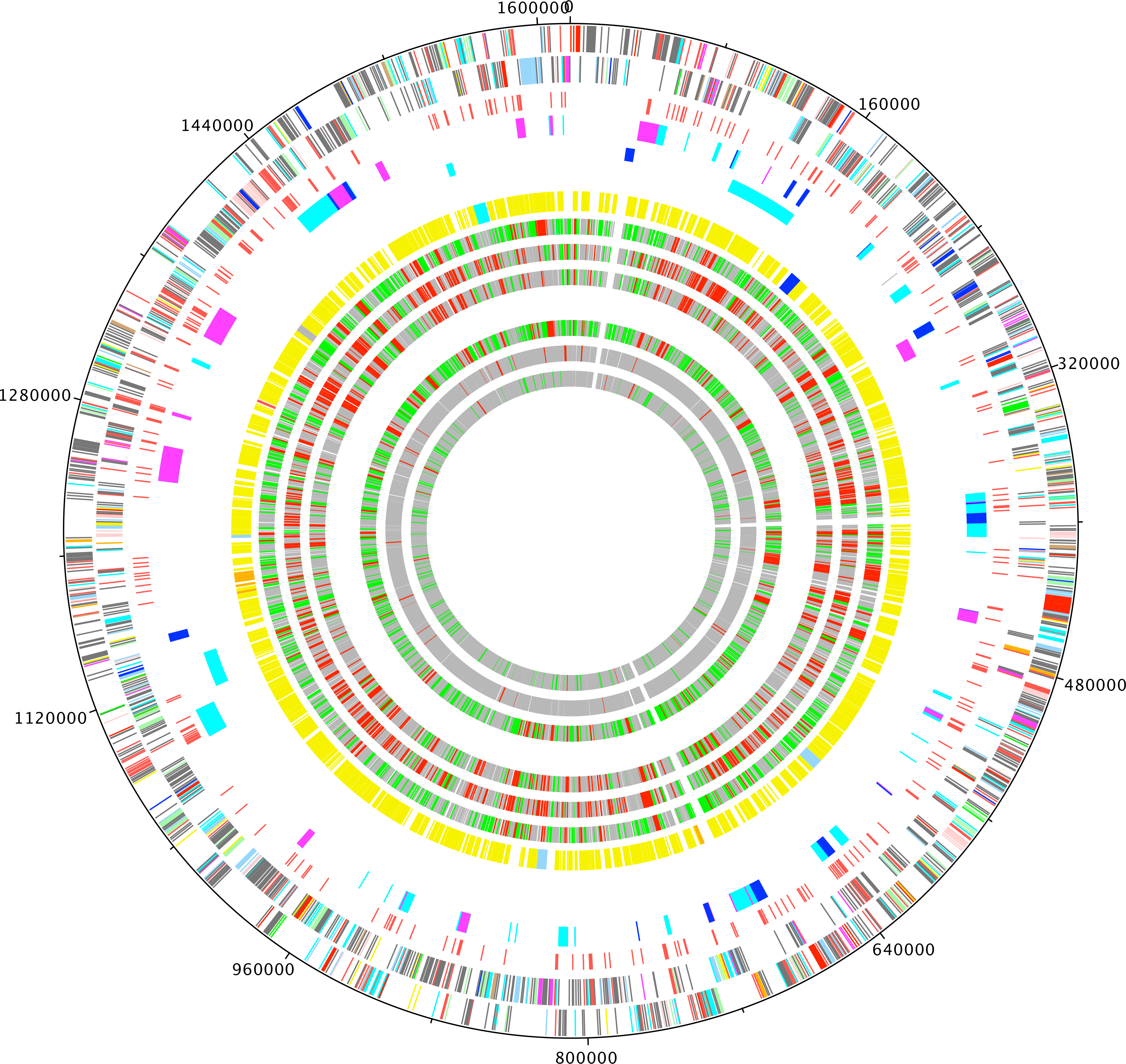

Supplement: S2 Fig — From the outside in: Track 1 C. jejuni M1 genome; Track 2 CDS forward strand; Track 3 CDS reverse strand; Track 4 hypothetical/ putative uncharacterized genes (CDS); Track 5 and 6 Regions of Difference (RODs) in the C. jejuni M1 genome compared to C. jejuni 414 (turquoise), M1 compared to NCTC11168 (magenta) and M1 compared to 1336 (blue); Track 7 operons in M1 as predicted by Rockhopper; Track 8 genes that are statistically significantly upregulated (green) or downregulated (red) at Time 0 only, compared to the Control; Track 9 genes that are statistically significantly upregulated (green) or downregulated (red) in 25°C (24 h) only compared to the Control; Track 10 genes that are statistically significantly upregulated (green) or downregulated (red) in 4°C (72 h) only compared to the Control; Track 11 genes that are statistically significantly upregulated (green) or downregulated (red) in 25°C (24 h) only compared to Time 0; Track 12 genes that are statistically significantly upregulated (green) or downregulated (red) in 4°C (72 h) only compared to Time 0; Track 13 genes that are statistically significantly upregulated (green) or downregulated (red) in 4°C (72 h) only compared to 25°C (24 h). Colouring of Tracks 2 and 3: replication related = bright red; efflux pumps = bright green; chemotaxis = bright blue; hydrogenases = pale pink; iron or heme-related = light brown; hydrogenases = rose; chaperone = orange; lipoproteins = yellow; membrane or periplasmic proteins = turquoise; ATP-/ ABC transporters = light green; ribosomal/RNA/ribonuclease = light blue; flagellar-related = magenta; hypotheticals = salmon. (TIFF) [file pone.0188936.s008.tiff]
